# The diverse magneto-optical selection rules in bilayer black phosphorus

**DOI:** 10.1038/s41598-018-31358-w

**Published:** 2018-09-05

**Authors:** Jhao-Ying Wu, Szu-Chao Chen, Thi-Nga Do, Wu-Pei Su, Godfrey Gumbs, Ming-Fa Lin

**Affiliations:** 1 Center of General Studies, National Kaohsiung Marine University, Kaohsiung, 811 Taiwan; 2 Department of Physics, National Cheng Kung University, Tainan, 701 Taiwan; 3 Department of Physics, National Kaohsiung Normal University, Kaohsiung, Taiwan; 4 Department of Physics, University of Houston, Houston, Texas USA; 5 Department of Physics and Astronomy, Hunter College at the City University of New York, New York, 10065 USA; 6 Donostia International Physics Center (DIPC), Paseo de Manuel Lardizabal 4, 20018 San Sebastían Donostia, Spain; 7 Hierarchical Green-Energy Materials/quantum topology centers, Tainan, 701 Taiwan

## Abstract

The magneto-optical properties of bilayer phosphorene is investigated by the generalized tight-binding model and the gradient approximation. The vertical inter-Landau-level transitions, being sensitive to the polarization directions, are mainly determined by the spatial symmetries of sub-envelope functions on the distinct sublattices. The anisotropic excitations strongly depend on the electric and magnetic fields. A uniform perpendicular electric field could greatly diversify the selection rule, frequency, intensity, number and form of symmetric absorption peaks. Specifically, the unusual magneto-optical properties appear beyond the critical field as a result of two subgroups of Landau levels with the main and side modes. The rich and unique magnetoabsorption spectra arise from the very close relations among the geometric structures, multiple intralayer and interlayer hopping integrals and composite external fields.

## Introduction

Phosphorene, a two-dimensional allotrope of the group-V element black phosphorus (BP), has recently attracted many theoretical and experimental studies. Layered phosphorene presents the puckered configuration, which is different to the planar structure in graphene. It is closely related to the *sp*^3^ hybridization of four (3*s*, 3*p*_*x*_, 3*p*_*y*_, 3*p*_*z*_) orbitals in BP. Furthermore, the deformed hexagonal lattice in the x-y plane is quite different from the honeycomb lattice of group-IV systems^1^. This unique geometric structure fully dominates the low-lying energy bands which are highly anisotropic in the dispersion relations as a function of the in-plane wave vector **k**, i.e., the linear and parabolic dispersions near the Fermi energy *E*_*F*_, respectively, along the ${\hat{k}}_{x}$ and ${\hat{k}}_{y}$ directions^1^. The carrier effective masses are anisotropic and asymmetric between electrons and holes^2,3^. Accordingly, the best functional performances in nanoelectronics and optoelectronics depend on the rotation-induced orientations of BP systems, which could be deduced from optical spectroscopy measurements^3^. The strong anisotropy sets phosphorene aside from most 2D materials, such as graphene, boron nitride and transition metal dichalcogenides.

Up to now, phosphorene has been successfully synthesized by various experimental techniques, covering mechanical cleavage^4–6^, liquid exfoliation^7–10^, mineralizer-assisted short-way transport reaction^11–13^, growing on red phosphorus (RP)^14^, converting from RP^15^, pulsed laser deposition^16^ and plasma thinning^17^. A layer-dependent energy gap of ~0.5–2 eV^1,18,19^ in phosphorene has been verified by optical experiments^5,20^. Such band gaps are larger than that of its bulk counterpart (~0.3 eV)^4,18,21^ and are in sharp contrast with the zero or narrow gaps of two-dimensional (2D) group-IV materials^22^. Transport measurements have demonstrated that the phosphorene-based field-effect transistor exhibits an on/off ratio of 10^5^ and the carrier mobility at room temperature as high as 10^3^ cm^2^/V⋅s^4^. The high performance implies potential applications in nanoelectronics. Specifically, many remarkable anisotropic physical phenomena are reported, such as mechanical strains^23^, excitonic effects^2^, optical spectra^4^; charge transport^24–26^ and thermal^27^ properties. The peculiar characteristics can be traced back to its feature-rich lattice structure and electronic properties.

Phosphorene exhibits unusual optical properties, such as high optical absorption in the UV region and dichroism. The threshold absorption structure, corresponding to the band-gap energy, is revealed with light polarization along the armchair direction^3^. Its frequency falls off rapidly with the number of layers. In contrast, the band-gap absorption is forbidden under the zigzag-direction electric polarization, mainly owing to the specific symmetry of wave functions in the initial and final states. Theoretically speaking, the electric dipole excitations, which connect the valence and conduction band states, are completely different for two perpendicular polarization directions^3^. The strong anisotropy is also predicted in the magneto-optical conductivity of BP thin films^28^. Interestingly, the essential physical properties of BP could be greatly diversified by a uniform electric field (*E*_*z*_$\hat{z}$)^29,30^. *E*_*z*_$\hat{z}$ might create a gapless band structure after reaching a critical value (*E*_*z*,*c*_) or induce various energy bands such as parabolic bands, graphene-like Dirac cones and oscillatory bands. This arises from a competitive or cooperative relation between the intralayer and interlayer atomic interactions and the Coulomb potential energy. The strong electrically tunable energy bands enrich the quantization phenomena under a uniform perpendicular magnetic field (*B*_*z*_$\hat{z}$), including two subgroups of Landau levels (LLs), uniform and non-uniform LL energy spacings and frequent crossings and anti-crossings^31^. There exist dramatic changes in the sub-envelope functions for the two mixed LLs with anti-crossing behaviors.

Motivated by the above-mentioned works, here we investigate the tuning effects, brought about by a perpendicular electric field, on the magneto-optical absorption spectra of bilayer phosphorene. The relations among the geometric structure, intrinsic interactions and external fields are explored in detail. The generalized tight-binding model^32^ has been developed to investigate the electronic properties of monolayer and bilayer phosphorus in a composite electric and magnetic field. The electric-dipole transition elements are evaluated within the gradient approximation^33,34^. There exists certain challenges in performing the numerical calculations of the magneto-absorption spectra of bilayer phosphorene. Since the magnetic Hamiltonian is a very huge matrix within achievable experimental field strengths (details in the Methods section), a long period of time is needed for solving the eigenvalues and eigenvectors. Moreover, the matrix elements of the velocity operators (Eq. (3) in the Methods section) are hard to compute. To overcome these challenges, we utilize a band-like matrix^32^ and the strong localization features of Landau wavefunctions to efficiently derive the absorption spectra. In addition, we use the method of gradient approximation to evaluate the matrix elements of the velocity operators as shown in Eq. (4).

**Figure 1:**
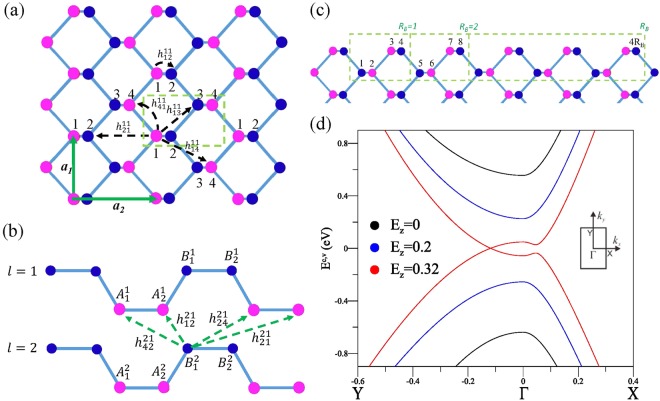
Geometric structures of bilayer phosphorene for the (**a**) top view and (**b**) side view with various intralayer and interlayer atomic interactions. In the presence of a uniform perpendicular magnetic field, an enlarged unit cell is in rectangular shape as shown in (**c**). The band structures under various electric fields are presented in (**d**).

**Figure 2:**
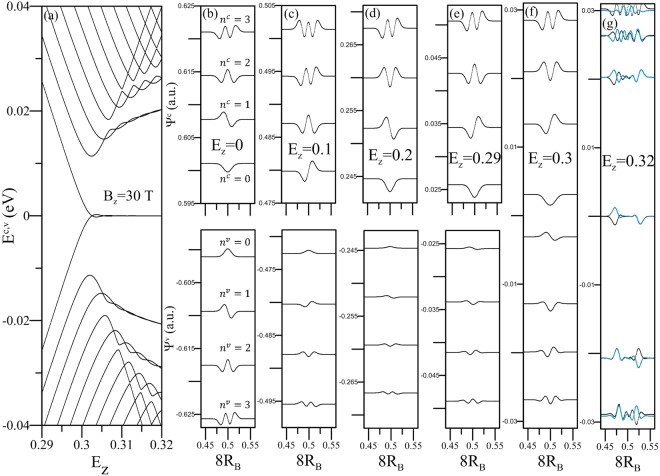
The (**a**) *E*_*z*_-dependent LL spectrum at *B*_*z*_ = 30 T and the (**b**–**g**) corresponding wavefunctions at various *E*_*z*_’s.

**Figure 3:**
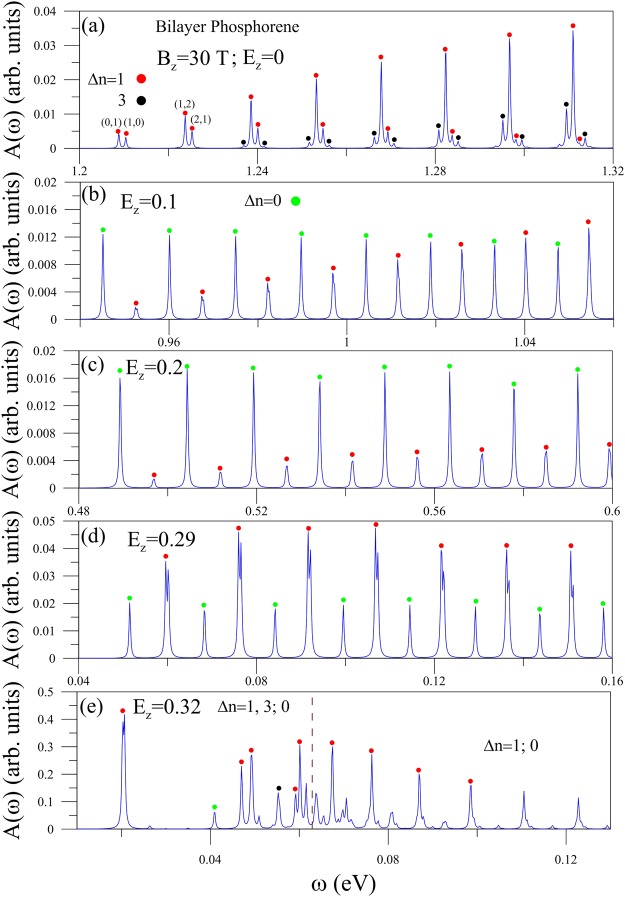
The *x*-polarized magneto-optical absorption spectra at *B*_*z*_ = 30 T for various electric-field strengths of (**a**) *E*_*z*_ = 0, (**b**) 0.1, (**c**) 0.2, (**d**) 0.29 and (**e**) 0.32 (V/Å). The pairs of numbers in (**a**) indicate the excitation channels.

**Figure 4:**
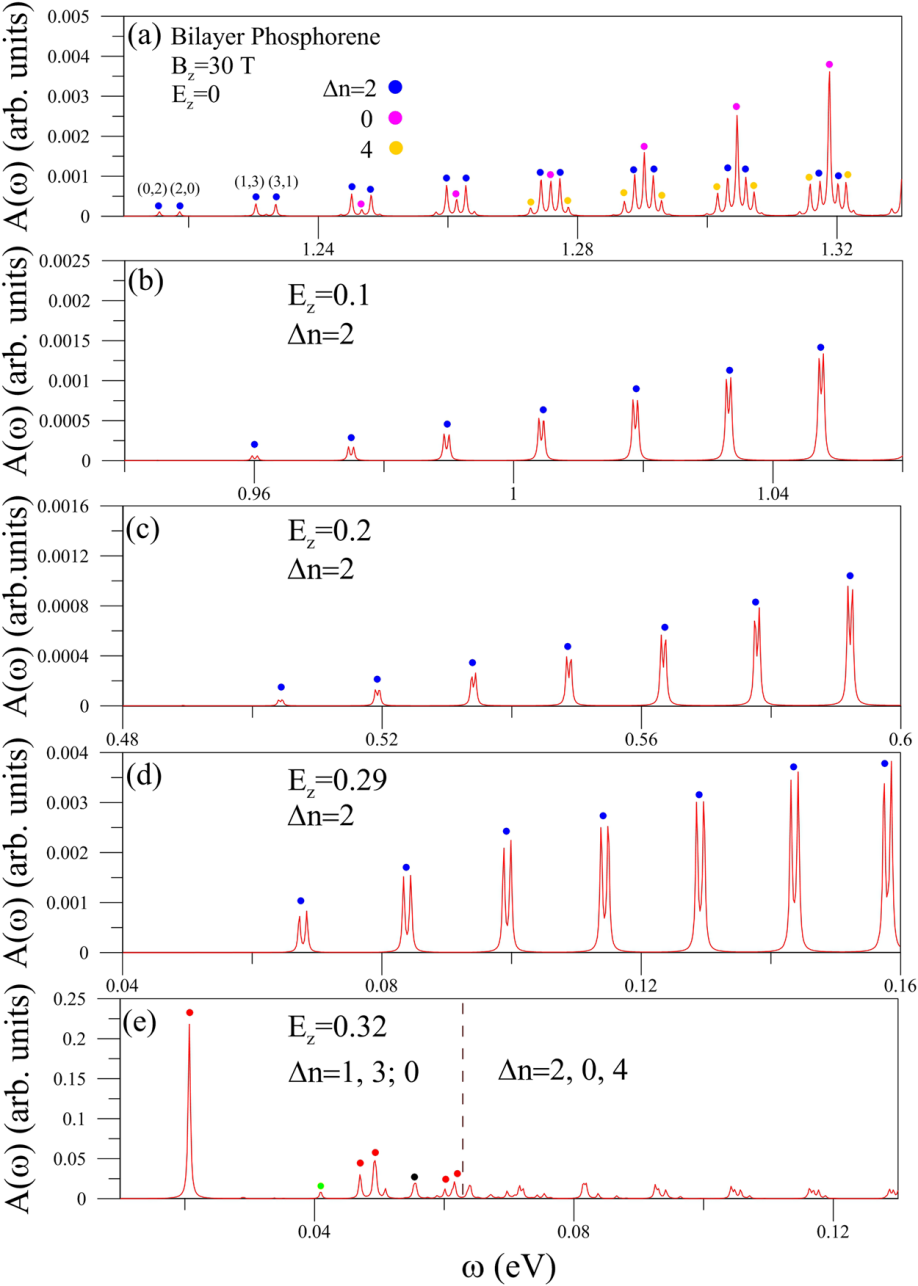
Similar plot as Fig. 3 for the *y*-polarization.

**Figure 5:**
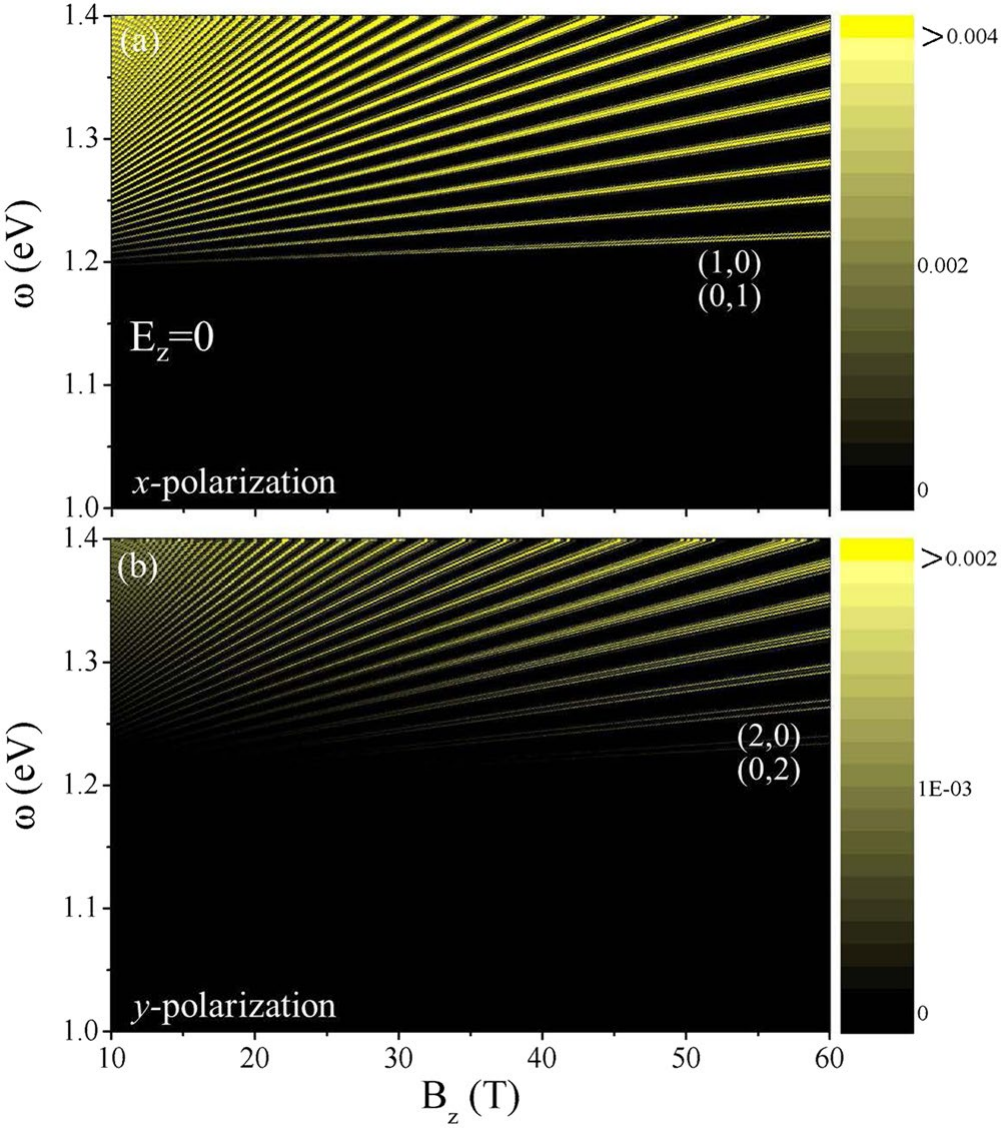
The *B*_*z*_-dependent optical absorption frequency at *E*_*z*_ = 0 under (**a**) *x*- and (**b**) *y*-polarizations. The threshold excitation channels are indicated by the pairs of numbers. The color scale represents the intensity of the energy-loss function.

**Figure 6:**
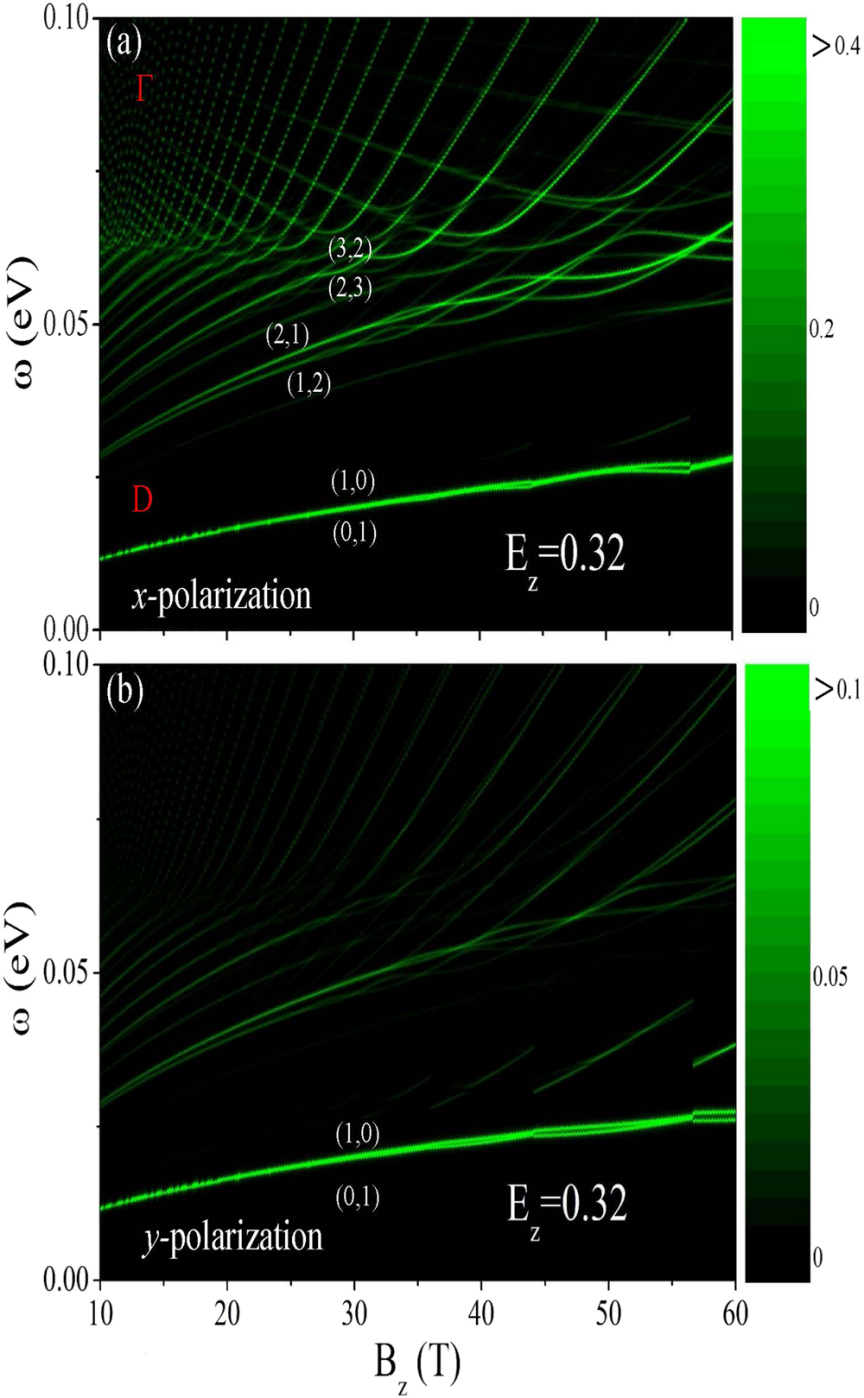
Same plot as Fig. 5, but shown at *E*_*z*_ = 0.32. The lowest few excitation channels in (**a**) are marked by the pairs of numbers.

**Figure 7:**
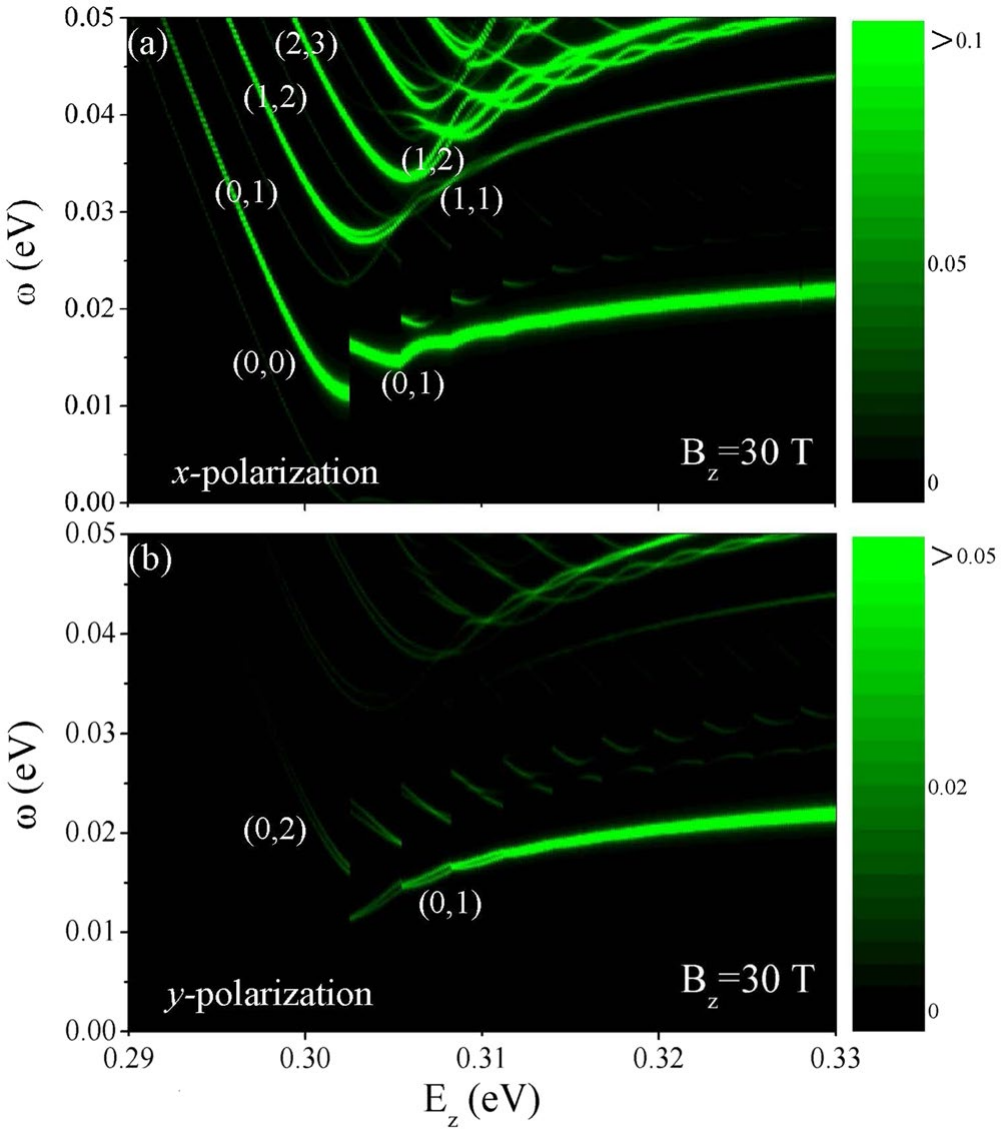
The *E*_*z*_-dependent absorption frequency at *B*_*z*_ = 30 T under (**a**) *x*- and (**b**) *y*-polarizations. The color scale represents the intensity of the energy-loss function.

In order to thoroughly explore the magneto-optical properties which significantly reflect the main features of LLs, we first present a simple review of magneto-electronic properties for which we refer to Figs 1 and 2^31^. Moreover, the effects of an electric field are demonstrated by the *E*_*z*_-dependent LL wavefunctions (Fig. 2(b–g)). The magneto-optical absorption spectra are predicted to be strongly dependent on the polarization directions and the electric- and magnetic-field strengths (demonstrated in Figs 3–7). Through careful calculations and detailed analysis, the available inter-LL transitions are deduced to be mainly determined by the spatial distributions/symmetries of sub-envelope functions on the distinct sublattices. The selection rule, frequency, intensity, number and structure of absorption peaks are dramatically changed by the *E*_*z*_ field. Whether the usual/unusual optical properties are revealed in the magneto-absorption spectra relies on the electric field beyond the critical one or not. This is well explained by exploring the diverse selection rules. These unique phenomena, occurred in the near to mid-infrared region, might be important for a wide range of optical technologies, such as spectroscopy, materials processing and chemical and biomedical sensing. They result from the peculiar geometric structures, multiple intralayer and interlayer hopping integrals and composite external fields. Such factors are incorporated in our framework simultaneously, meaning that the methodology is readily extended to other low-dimensional systems with arbitrary layer number and stacking structure. The predicted results could be validated by magneto-optical measurements^35–42^.

## Methods

In a single layer of phosphorene, the P atoms lie on the lower (pink circles) and higher (blue circles) planes due to the puckered honeycomb structure, as shown in Fig. 1(a,b). The four atoms in a primitive unit cell (as marked by the dashed green lines in Fig. 1(a)) belong to four different sublattice sites, denoted as *A*_1_, *A*_2_, *B*_1_ and *B*_2_. For a N-layer system, there are 4 × N atoms in a unit cell, e.g., 8 atoms for bilayer phosphorene (Fig. 1(b)). The low-lying energy bands are mainly dominated by the atomic interactions of 3 *p*_*z*_ orbitals. The Hamiltonian of a few-layer phosphorene can be expressed as

1 $$H=\sum _{l}\sum _{i=1}^{4}\,({\varepsilon }_{i}^{l}+{U}_{i}^{l}){c}_{i}^{l}{c}_{i}^{\dagger l}+\sum _{l,\langle i,j\rangle }\,{h}_{ij}^{ll}{c}_{i}^{l}{c}_{j}^{\dagger l}+\sum _{l\ne l^{\prime} ,\langle i,j\rangle }\,h{^{\prime} }_{ij}^{ll^{\prime} }{c}_{i}^{l}{c}_{j}^{\dagger l^{\prime} }.$$

Here, ${\varepsilon }_{i}^{l}\,\mathrm{=1}$ eV is a layer- and sublattice-dependent site energy due to the chemical environment difference between the A and B sublattices in bilayer BP^1^. ${U}_{i}^{l}$ is the Coulomb potential energy induced by an electric field. They both contribute to the diagonal matrix elements. The summation covers all the lattice sites on two layers. ${c}_{i}^{l}$ $({c}_{j}^{\dagger l^{\prime} })$ is the annihilation (creation) operator which can destroy (create) an electronic state on the *i*-th (*j*-th) lattice site of the *l*-th (*l*′-th) layer; *i* = 1, 2, 3, 4, respectively, correspond to the P atoms on the *A*_1_, *B*_1_, *B*_2_ and *A*_2_ sublattices. ${h}_{ij}^{ll}$ and $h{^{\prime} }_{ij}^{ll^{\prime} }$ represent the intralayer and interlayer hopping integrals, respectively. The effective atomic interactions used in the calculations are denoted by ${h}_{12}^{11}$ = 3.665 eV, ${h}_{21}^{11}=-\,0.055$ eV, ${h}_{13}^{11}=-\,0.105$ eV, ${h}_{14}^{11}=-\,0.205$ eV, ${h}_{41}^{11}=-\,1.22$ eV, ${h}_{12}^{21}=0.295$ eV, ${h}_{21}^{21}=-\,0.091$ eV, ${h}_{24}^{21}=-\,0.151$ eV and ${h}_{42}^{21}=0.273$ eV^1^, as indicated in Fig. 1(a,b).

When bilayer phosphorene is present in a uniform perpendicular magnetic field, the magnetic flux through a unit rectangle is Φ = *a*_1_*a*_2_*B*_*z*_, where *a*_1_ = 3.27 Å and *a*_2_ = 4.43 Å are lattice constants (the lattice vectors shown by the green arrows in Fig. 1(a)). The vector potential, $\overrightarrow{A}$ = (*B*_*z*_*x*)$\hat{y}$, can create the Peierls phase of $exp\{i[\frac{2\pi }{{\varphi }_{0}}\int \overrightarrow{A}\cdot d\overrightarrow{r}]\}$ in the hopping integrals, leading to a new period along $\hat{x}$ and thus an enlarged rectangular unit cell with 4*R*_*B*_ = 4*ϕ*_0_/Φ atoms in each phosphorene layer, as illustrated in Fig. 1(c). *ϕ*_0_ ( = hc/e = 4.1 × 10^−15^ T⋅m^2^) is the magnetic flux quantum; *ϕ*_0_/Φ is chosen to be an integer for a model study. The reduced first Brilloun zone has an area of 4*π*^2^/*a*_1_*a*_2_*R*_*B*_, in which all the LLs are degenerate for any (*k*_*x*_, *k*_*y*_) states. For bilayer BP, the magnetic Hamiltonian matrix, which is built from the 8*R*_*B*_ tight-binding functions, has 8*R*_*B*_ × 8*R*_*B*_ elements. This Hamiltonian is a huge Hermitian matrix within achievable experimental field strengths, e.g., the dimension reaches 50400 at *B*_*z*_ = 10 T.

After the diagonalization of bilayer magnetic Hamiltonian, the LL wave function, with quantum number *n*, could be expressed as

2 $$\psi (n,{\bf{k}})=\sum _{l=\mathrm{1,2}}\,\sum _{\alpha =\mathrm{1,2}}\,\sum _{\beta =1}^{{R}_{B}}\,[{A}_{\alpha ,\beta }^{l}(n,{\bf{k}})|{\varphi }_{\alpha ,\beta }^{l}(A)\rangle +{B}_{\alpha ,\beta }^{l}(n,{\bf{k}})|{\varphi }_{\alpha ,\beta }^{l}(B)\rangle ],$$

where ${\varphi }_{\alpha \,,\beta }^{l}$ is the tight-binding function localized at the sublattice-dependent lattice sites. ${A}_{\alpha \,,\beta }^{l}(n\,,{\bf{k}})$/${B}_{\alpha \,,\beta }^{l}(n\,,{\bf{k}})$ is the amplitude on the $({A}_{1}^{l},{A}_{2}^{l},{B}_{1}^{l},{B}_{2}^{l})$-sublattice-dependent lattice site. Specifically, all the amplitudes in an enlarged unit cell could be regarded as the spatial distributions of the sub-envelope functions on the distinct sublattices. Bilayer BP has eight magnetic sub-envelope functions, in which the dominating ones are utilized to characterize the magnetic quantum numbers and the types of LLs. They provide much information for explaining the peculiar LL behaviors, as discussed below. The strong electrically-tunable LL energy spectra exist when *B*_*z*_ < 60 T. The theoretical framework incorporates the intra-layer and inter-layer atomic interactions, as well as the effects due to geometric structures and external fields, simultaneously. It is suitable for studying the diverse quantization phenomena in layered materials with distinct stacking configurations under uniform/modulated/composite external fields. Moreover, the calculated results are accurate and reliable over a wide energy range.

The electric-dipole optical excitations directly reflect the main characteristics of the electronic properties. The occupied valence LL of *n*^*v*^ is vertically excited to the unoccupied conduction LL of *n*^*c*^ in the presence of an electromagnetic wave at zero temperature, being characterized by the excitation channel of Δ*n* = |*n*^*v*^−*n*^*c*^|. Based on the linear-response Kubo theory, the optical spectral function, without the change of wave vector in the inter-LL transition, is given by

3 $$\begin{array}{ccc}A(\omega ) & \propto  & 4(4{\pi }^{2}/{a}_{1}{a}_{2}{R}_{B})\,\sum _{{n}^{c},{n}^{v}}\,{|\langle {\psi }^{c}({n}^{c},{\bf{k}})|\frac{\hat{{\bf{E}}}\cdot {\bf{p}}}{m}|{\psi }^{v}({n}^{v},{\bf{k}})\rangle |}^{2}\\  &  & \times \frac{\gamma }{{[{E}^{c}({n}^{c},{\bf{k}})-{E}^{v}({n}^{v},{\bf{k}})-\omega ]}^{2}+{\gamma }^{2}}\mathrm{.}\end{array}$$

The contributions, which come from the spin, localization-center and **k** degeneracies, are included in the first factor appearing before the summation. The available transition channels and the excitation intensity depend on the square of the velocity matrix element |*M*(*n*^*c*^, *n*^*v*^)|^2^. The last factor is the joint density-of-states with the broadening parameter *γ*, which is related to the initial [(*n*^*v*^, **k**)] and final [(*n*^*c*^, **k**)] LLs. Also, $\hat{{\bf{E}}}$ is a unit vector of electric polarization and **p** is momentum. The parallel and perpendicular polarizations, $\hat{{\bf{E}}}||\hat{{\bf{x}}}$ and $\hat{{\bf{E}}}\perp \hat{{\bf{x}}}$, are taken into account, respectively. The velocity matrix element *M*(*n*^*c*^, *n*^*v*^) is calculated within the gradient approximation and is expressed by the amplitudes ${A}_{\alpha ,\beta }^{l}$’s(${B}_{\alpha ,\beta }^{l}$’s) of the tight-binding functions $({\varphi }_{\alpha ,\beta }^{l})$’s on the distinct sublattices. For $\hat{{\bf{E}}}||\hat{{\bf{x}}}$, the velocity matrix element is evaluated from

4 $$\begin{array}{rcl}M({n}^{c},{n}^{v}) & = & \sum _{l,l^{\prime} =\mathrm{1,2}}\,\sum _{\alpha ,\alpha ^{\prime} =\mathrm{1,2}}\,\sum _{\beta ,\beta ^{\prime} =1}^{{R}_{B}}\,\{{A}_{\alpha ^{\prime} ,\beta ^{\prime} }^{\ast l^{\prime} }({n}^{v},{\bf{k}})\times {A}_{\alpha ,\beta }^{l}({n}^{c},{\bf{k}})\langle {\varphi }_{\alpha ^{\prime} ,\beta ^{\prime} }^{l^{\prime} }(A)|\frac{{p}_{x}}{m}|{\varphi }_{\alpha ,\beta }^{l}(A)\rangle \\  &  & +\,{A}_{\alpha ^{\prime} ,\beta ^{\prime} }^{\ast l^{\prime} }({n}^{c},{\bf{k}})\times {B}_{\alpha ,\beta }^{l}({n}^{v},{\bf{k}})\langle {\varphi }_{\alpha ^{\prime} ,\beta ^{\prime} }^{l^{\prime} }(A)|\frac{{p}_{x}}{m}|{\varphi }_{\alpha ,\beta }^{l}(B)\rangle \\  &  & +\,{B}_{\alpha ^{\prime} ,\beta ^{\prime} }^{\ast l^{\prime} }({n}^{c},{\bf{k}})\times {A}_{\alpha ,\beta }^{l}({n}^{v},{\bf{k}})\langle {\varphi }_{\alpha ^{\prime} ,\beta ^{\prime} }^{l^{\prime} }(B)|\frac{{p}_{x}}{m}|{\varphi }_{\alpha ,\beta }^{l}(A)\rangle \\  &  & +\,{B}_{\alpha ^{\prime} ,\beta ^{\prime} }^{\ast l^{\prime} }({n}^{c},{\bf{k}})\times {B}_{\alpha ,\beta }^{l}({n}^{v},{\bf{k}})]\langle {\varphi }_{\alpha ^{\prime} ,\beta ^{\prime} }^{l^{\prime} }(B)|\frac{{p}_{x}}{m}|{\varphi }_{\alpha ,\beta }^{l}(B)\rangle \}.\end{array}$$

In the gradient approximation, $\langle {\varphi }_{\alpha ^{\prime} ,\beta ^{\prime} }^{l^{\prime} }|\frac{{p}_{x}}{m}|{\varphi }_{\alpha ,\beta }^{l}\rangle \cong \frac{\partial }{\partial {k}_{x}}\langle {\varphi }_{\alpha ^{\prime} ,\beta ^{\prime} }^{l^{\prime} }|H|{\varphi }_{\alpha ,\beta }^{l}\rangle$, depending on the intralayer and interlayer hopping integrals combined with the Peierls phases. The quantity *M*(*n*^*c*^, *n*^*v*^), which is determined by the relations among eight sub-envelope functions and the sublattice-dependent hopping integrals/Peierls phases, can account for the magneto-optical selection rules. By accurate calculations and detailed examinations of the well-behaved LLs, the product of three terms in Eq. (4) remains the same or changes the sign after the interchange of the initial and final states on the sublattices. Its direct summation leads to a finite (vanishing) velocity matrix element for Δ*n* = odd (even) integers under the *x*-polarization (discussed latter for Fig. 3) and the opposite behavior is revealed for the *y*-polarization (Fig. 4).

## Results and Discussion

### Landau Level Spectrum

The special lattice structure and complicated hopping integrals generate rich band structures. Bilayer BP has a direct gap of *E*_*g*_ ~ 1 eV near the Γ point, as illustrated in Fig. 1(d) by the black curves, being smaller than that (~1.6 eV) for the monolayer system. The highly anisotropic energy bands yield the approximately linear and parabolic dispersions along Γ X and Γ Y (${\hat{k}}_{x}$ and ${\hat{k}}_{y}$), respectively. The energy gap could be reduced considerably by applying an electric field (blue curves). The semiconductor-semimetal transition appears at the strength larger than ${E}_{z,c}\simeq 0.3$ (V/Å; red curves), for which the valence and conduction bands are transformed into linearly intersecting bands and oscillatory bands along Γ Y and Γ X, respectively. Two split Dirac-cone structures are situated on the right- and left-hand sides of the Γ point (along $+{\hat{k}}_{y}$ and $-{\hat{k}}_{y}$). The extreme points remain at the Γ point, accompanied by two saddle points on the opposite ${k}_{x}^{^{\prime} }s$. The electronic states near the Dirac and Γ points will be magnetically quantized into two distinct LL subgroups (discussed in Fig. 2)^31^.

All the critical points and constant-energy loops in the energy-wave vector space will dominate the main features of LLs. When *E*_*z*_ = 0, each LL is four-fold degenerate for each (*k*_*x*_, *k*_*y*_) state in the presence of spin and localization-center degeneracies. The occupied LLs are asymmetric compared with the unoccupied ones near the Fermi level. The LL energy spectrum is very sensitive to the electric-field strength, as illustrated in Fig. 2(a). The conduction and valence LL energies, respectively, rapidly decline and grow with an increase of *E*_*z*_. The lowest unoccupied and the highest occupied LLs, corresponding to band-edge states, merge together at *E*_*z*,*c*_, in which they are closely related to the magnetic quantization of electronic states near the Dirac points. A larger *E*_*z*_ can extend the range of linear energy bands and thus double the degeneracy of low-lying LLs (from two neighboring Dirac points in ref.^31^). The LL anti-crossings and crossings occur frequently, since there are two competitive/cooperative LL subgroups initiated from the Dirac and Γ points, respectively^31^.

The amplitude, localization center and oscillation mode of the LL wave functions strongly depend on the electric-field strength. For bilayer BP systems, the eight sub-enevlope functions on the distinct sublattices have simple relations. Therefore, ${{\rm{\Psi }}}^{c,v}\,({A}_{1}^{1})$ is illustrated to understand the critical characteristics and the *E*_*z*_-dependence. In the absence of *E*_*z*_, ${{\rm{\Psi }}}^{c}({A}_{1}^{1})=-\,{{\rm{\Psi }}}^{c}({A}_{2}^{1})={{\rm{\Psi }}}^{c}({A}_{1}^{2})=-\,{{\rm{\Psi }}}^{c}({A}_{2}^{2})={{\rm{\Psi }}}^{c}({B}_{2}^{2})={{\rm{\Psi }}}^{c}({B}_{2}^{1})=-\,{{\rm{\Psi }}}^{c}({B}_{1}^{2})=-\,{{\rm{\Psi }}}^{c}({B}_{1}^{1})$ and ${{\rm{\Psi }}}^{v}({A}_{1}^{1})={{\rm{\Psi }}}^{v}({A}_{2}^{1})={{\rm{\Psi }}}^{v}({A}_{1}^{2})={{\rm{\Psi }}}^{v}({A}_{2}^{2})=-\,{{\rm{\Psi }}}^{v}({B}_{2}^{2})=-\,{{\rm{\Psi }}}^{v}({B}_{2}^{1})=-\,{{\rm{\Psi }}}^{v}({B}_{1}^{2})=-\,{{\rm{\Psi }}}^{v}({B}_{1}^{1})$. The quantum number *n*^*c*^ (*n*^*v*^) for each conduction (valence) LL is clearly identified from the number of zero points. For example, the four low-lying conduction/valence LLs have *n*^*c*,*v*^ = 0, 1, 2; 3, as shown in Fig. 2(b). The localization centers are near the 1/2 and 2/2 positions of the enlarged unit cell of the crystal lattice, reflecting the magnetic quantization associated with the Γ point. The former is sufficient for studying the magneto-optical properties. The well-behaved spatial distributions are similar to those in monolayer graphene^43^. In addition, the deeper/higher *n*^*c*,*v*^ LLs might have the side modes of *n*^*c*,*v*^ ± 1, 2; 3 due to the complicated interlayer hopping integrals.

A perpendicular electric field breaks the inversion symmetry, leading to the probability transfer among the distinct sublattices and even the changes of spatial oscillation modes. With a gradual increase of *E*_*z*_, the four sub-envelope functions on the first and second layers, respectively, have a simple relation, as revealed in the *E*_*z*_ = 0 case (Fig. 2(b–d)). Such functions change slowly for the conduction LLs, while the opposite is true for the valence LLs. Their oscillation modes are dramatically changed from *n*^*v*^ into *n*^*v*^ + 1 as *E*_*z*_ gets close to the critical value, e.g., *E*_*z*_ = 0.29 and 0.3 (Fig. 2(e,f)). And then, the *n*^*c*^ = 0 and *n*^*v*^ = 1 LLs are hybridized with each other, so that their wavefunctions present the combination of two oscillation modes (*E*_*z*_ = 0.32 in Fig. 2(g)). Furthermore, there exist two localization centers on the left- and right-hand sides of the 1/2 position, reflecting the existence of two neighboring Dirac-cone structures^31^. The unusual/perturbed oscillation mode, with a main mode and significant side modes, are also revealed in other low-lying LLs. In addition, the LL splitting will become obvious at deeper/higher energies, in which the Γ-induced LL subgroup comes to exist and exhibits anti-crossing behaviors with the Dirac-point-related one. The various features of sub-envelope functions under distinct electric-field strengths imply the electrically tunable optical selection rules.

### Magneto-absorption spectra

The magneto-optical excitations directly reflect the main features of magnetic quantization. The available inter-LL excitation channels, which arise from transitions from the occupied *n*^*v*^ states to the unoccupied *n*^*c*^ ones, are denoted as (*n*^*v*^, *n*^*c*^). They exhibit a lot of delta-function-like absorption peaks, being sensitive to external fields and polarization directions. For the parallel polarization direction, the magneto-absorption symmetric peaks, as shown in Fig. 3(a) at *B*_*z*_ = 30 T, are dominated by the selection rules of Δ*n* = 1 and 3. The threshold peak comes from the (0, 1) channel. There are many double-peak structures as a result of the asymmetric LL energy spectrum (Fig. 2(a)). Furthermore, the Δ*n* = 1 absorption structures (red circles) might be much higher than the Δ*n* = 3 ones (black circles). Our numerical calculations show that the finite velocity matrix elements related to the former are induced by the symmetric/anti-symmetric superpositions of eight sub-envelopes in the initial and final LL states and the dominating intralayer hopping integral (${h}_{12}^{11}$) (Eq. 4). The extra selection rule, such as the latter, is driven by the side modes of the deeper/higher perturbed LLs under the interlayer hopping integrals. Specifically, all the inter-LL absorption peaks present non-uniform intensities, regardless of the selection rules.

The complex relations between the Coulomb potential energies and the intrinsic interactions might dramatically change the magneto-optical selection rules. In addition to the Δ*n* = 1 excitation channels, the Δ*n* = 0 ones (green circles) come to exist quickly as *E*_*z*_ gradually grows, as clearly demonstrated in Fig. 3(b) for *E*_*z*_ = 0.1. These two kinds of absorption peaks appear alternatively, in which the neighboring two peaks of the former are merged together and the threshold peak arises from the latter. The Δ*n* = 0 selection rule reflects the fact that an electric field could create an obvious asymmetry between the valence and conduction sub-envelope amplitudes on the same sublattices (Fig. 2(b)), especially for the low-lying LLs. This asymmetry also affects the peak intensities, so that the Δ*n* = 0 peaks are stronger (weaker) than the Δ*n* = 1 ones at lower (higher) frequency. The Δ*n* = 0 channels will become the dominant excitations with the further increase of field strength, e.g., *E*_*z*_ = 0.2 in Fig. 3(c), a result of the enhanced amplitude asymmetry (Fig. 2(d)). But, as *E*_*z*_ approaches the critical field, Δ*n* = 1 peaks increase rapidly, compared with the Δ*n* = 0 ones, e.g., *E*_*z*_ = 0.29 in Fig. 3(d). This is attributed to the *E*_*z*_-induced significant side modes in the low-lying valence LLs (Fig. 2(e)). Specially, for *E*_*z*_ ≥ *E*_*z*,*c*_, the Dirac-point-created LLs exhibit very strong absorption spectra at lower frequency, e.g., the prominent peaks of Δ*n* = 1, 3; 0 at *ω* < 0.06 eV under *E*_*z*_ = 0.32 (the left region of the brown vertical dashed line in Fig. 3(e)). The main reason is that such LLs have double degeneracy and two localization centers with the main and side modes (Fig. 2(g)). Moreover, the Γ-related LLs also make important contributions to the higher-frequency absorption peaks (the right region of the brown vertical dashed line).

The magneto-optical properties are strongly anisotropic even for the degenerate LLs. The spectral intensities in the perpendicular polarization decline significantly as indicated from the comparison between Figs 3(a) and 4(a). The *y*-polarization velocity matrix element depends on the smaller hopping integrals, but is independent of the largest one (Eq. (4)). This illustrates the drastic changes of peak intensities during the variation of polarization direction. The magneto-optical selection rules become Δ*n* = 2, 0; 4 (blue, pink; yellow circles; discussed in Eq. (4)). The Δ*n* = 1; 3 channels disappear by satisfying a specific relation in the two-sublattice-dependent velocity matrix element. The Δ*n* = 2 and 0 channels, respectively, dominate absorption peaks at lower and higher frequencies. Apparently, the (0, 2) channel creates the threshold peak. When the electric field is below its critical value, only Δ*n* = 2 peaks survive (Fig. 4(b–d)). The Δ*n* = 0 selection rule is greatly suppressed through the strong asymmetry of LL wave-function amplitudes. However, more available excitation channels come to exist for *E*_*z*_ ≥ *E*_*z*,*c*_, e.g., the Δ*n* = 1, 3; 0 selection rules at *E*_*z*_ = 0.32 in Fig. 4(e)). A similar phenomenon, the diversified selection rule, also appears in the *x*-polarization (Fig. 3(e)), directly reflecting the characteristics of the Dirac-point-induced neighboring LL subgroup and the strong crossings/anti-crossings with the Γ-created LL subgroup.

The dependencies of the magneto-absorption spectra on the magnetic- and electric-field strengths deserve a closer examination. In the absence of *E*_*z*_, the peak frequencies, corresponding to the selection rules (Δ*n* = 1; 3 in Fig. 5(a) and Δ*n* = 2, 0; 4 in Fig. 5(b)), rise with increasing *B*_*z*_. The *B*_*z*_-dependence deviates from the square-root and linear behaviors, being different from those for monolayer graphene and the 2D electron gas. This is closely related to the unusual band structure (Fig. 1(d)). There exist certain important differences between the parallel and perpendicular polarizations in terms of the intensity, frequency and number of absorption peaks. The largest intralayer hooping integral involved in the *x*-polarization excitations is responsible for the significant anisotropy in the absorption intensity. The threshold frequencies in the *x*- and *y*-polarization are, respectively, determined by Δ*n* = 1 and 2, so that optical gaps are lower under the former. The monotonic *B*_*z*_-dependence is also revealed in electric fields below the critical one.

On the other side, magneto-optical properties possess the unique *B*_*z*_-dependence beyond the critical electric field, as shown in Fig. 6(a,b) at *E*_*z*_ = 0.32. Two categories of inter-LL excitations, which arise from the Dirac-cone- and Γ-valley-related LL subgroups (Fig. 2(a)), respectively, contribute to lower- and higher-frequency absorption peaks. The LL energies of the former and the latter, respectively, rise and decline with an increase in magnetic field and so do their absorption peak frequencies. The *B*_*z*_-dependent absorption frequencies become non-monotonic and abnormal when the anti-crossings of two subgroups frequently appear at sufficiently high magnetic field (*B*_*z*_ > 30 T). Specifically, the lower absorption frequencies due to the Dirac-cone LLs present the special $\sqrt{{B}_{z}}$-dependence for *B*_*z*_ < 30 T, as observed in monolayer graphene^38,39,44^. The first absorption peak is induced by the occupied/unoccupied LL at the Fermi level and the nearest conduction/valence LL. Its intensity is strongest among all the absorption peaks. The threshold frequency is independent of polarization direction. Furthermore, it increases with *B*_*z*_ in the monotonic form.

The main features of the magneto-absorption spectra are very sensitive to the electric-field strength. For *E*_*z*_ < *E*_*z*,*c*_, the frequencies of absorption peaks decrease rapidly e.g., *E*_*z*_ < 0.302 in Fig. 7(a,b) at *B*_*z*_ = 30 T. On the other hand, the unusual magneto-optical properties are revealed in the range of *E*_*z*_ > *E*_*z*,*c*_. They present non-monotonic, oscillatory and discontinuous *E*_*z*_-dependencies. Since the quantum modes are altered by a sufficiently high electric field, the new/original excitation channels will appear/disappear. Some absorption peaks could only survive for certain ranges of *E*_*z*_’s. Obviously, the threshold channel is changed from (0, 0) into (0, 1) [(0, 2) into (0, 1)] near the critical electric field under the *x*-polarization (the *y*-polarization). This even leads to the abrupt change of threshold frequency.

The above-mentioned features of the magneto-absorption spectra could be examined by various optical spectroscopies, such as infrared transmission^35–40^ and Raman scattering spectroscopies^41,42^. Up to now, experimental measurements have confirmed the rich and diverse magneto-optical properties in graphene-related systems, e.g., Bernal graphite^35–37^, AB-stacked few-layer graphene^38,39^ and carbon nanotubes^40^. The low-lying absorption peaks in layered graphene have been identified as agreeing with the selection rule of Δ*n* = 1^38,39^, in which the square-root and linear dependencies of excitation frequencies on *B*_*z*_, respectively, are attributed to the monolayer- and bilayer-like characteristics. The coexistence behavior is also observed in AB-stacked graphite^35–37^. Moreover, the periodic Aharonov-Bohm effect has been verified to exist in cylindrical nanotube systems^40^. The magneto-optical properties in bilayer BP, the selection rule, number, frequency and intensity of absorption peaks, are greatly diversified/enriched by the polarization direction and external fields; that is, they are easily tuned by external factors. The experimental identifications are very useful in understanding the effects due to the geometric symmetry, intrinsic interactions; electric and magnetic fields. It should be noticed that bilayer BP is very different from AB- and AA-stacked graphene^45,46^ with respect to the anisotropy/isotropy, selection rules; *B*_*z*_- and *E*_*z*_-dependencies of magnet-optical spectra. In general, the latter possess almost isotropic excitations, a dominant selection rule with Δ*n* = 1 and a linear or square-root relation between *B*_*z*_ and frequency of absorption peak.

## Conclusions

The generalized tight-binding model, in conjunction with the Kubo formula, has been utilized to investigate the rich and unique magneto-optical properties of bilayer BP. The main features of the LLs, the field-dependent energy spectra and wavefunctions, are well characterized by the oscillation modes of sub-envelope functions on the distinct sublattices. They account for the selection rule, frequency and number of magneto-absorption peaks, strongly depending on the polarization directions; electric and magnetic fields. The predicted results could be verified by magneto-optical spectroscopies, as it has already been done for graphene-related systems^35–42^. The theoretical framework could be further developed to fully understand the diverse magnetic quantization phenomena, especially for the generalization to emergent 2D systems covering few-layer silicene, germanene, tinene, antomonene, bismuthene, etc.

Bilayer BP exhibits the highly anisotropic magneto-absorption spectra, various selection rules and the usual/abnormal field dependencies. The spectral intensity declines obviously during the variation from the *x*- to *y*-polarization. This is determined by whether the velocity matrix element is associated with the largest intralayer hopping integral. The selection rules, which come from the well-behaved LLs in the absence of *E*_*z*_, are characterized by Δ*n* = odd and even integers for the *x*- and *y*-polarizations, respectively. The absorption frequencies deviate from the linear or square-root *B*_*z*_-dependence. With the increase of *E*_*z*_, the spectral intensities of available channels might be greatly enhanced/suppressed, or the extra selection rules come to exist. The non-monotonic and oscillatory field dependencies are revealed in electric fields beyond the critical one, e.g., the drastic/dramatic changes in threshold frequency/channel near *E*_*z*,*c*_. Such magneto-optical properties clearly illustrate the close relations among the geometric symmetry, intralayer and interlayer atomic interactions and external fields. There exist important differences between bilayer BP and graphene in the main features of magneto-optical spectra.
